# Dataset on protein composition of a human plasma sub-proteome able to modulate the Dengue 2 virus infection in Huh 7.5 cells

**DOI:** 10.1016/j.dib.2015.12.016

**Published:** 2015-12-17

**Authors:** Vivian Huerta, Yassel Ramos, Alexis Yero, Dianne Pupo, Dayron Martin, Gabriel Márquez, Alejandro Martín, Mónica Sarría, Sebastien Gallien, Luis J. González, Bruno Domon, Glay Chinea

**Affiliations:** a Center for Genetic Engineering and Biotechnology (CIGB), Cuba; b Luxembourg Clinical Proteomics Center, Luxembourg

**Keywords:** Human plasma, Anion exchange chromatography, Dengue virus

## Abstract

The four serotypes of dengue virus (DENV1-4) are the causal agents of the emerging disease Dengue Fever and its severe forms. DENV is inoculated into human blood through a mosquito bite. Thus, plasma is an important media for DENV dissemination in infected persons and several important interactions should take place for the virus with human plasma proteins that strongly influence or may determine the course of the infection. This dataset contains 239 proteins identified in the elution fractions of human plasma subjected to DE-52 anion exchange chromatography. Data on DENV2 infection of Huh 7.5 cells in presence of the human plasma fraction is also presented.

## 1 Specifications table

**Table:** 

Subject area	*Biology*
More specific subject area	*Chromatographic fractionation of human plasma*
Type of data	*Figures, table and an excel file with the output of Thermo Scientific™ Proteome Discoverer™ software*
How data was acquired	*Mass spectrometry, linear trap quadrupole (LTQ)-Orbitrap Velos mass spectrometer*
Data format	*Processed*
Experimental factors	*Dengue infection under different Human plasma samples preparations*
Experimental features	*Human plasma was adjusted to pH 6.0 and subjected to anion exchange chromatography. Elution fractions were digested with trypsin and protein species were identified by peptide sequencing. Virology assays were performed to investigate the effect of plasma fractions on dengue virus infection to human cells.*
Data source location	*Center for Genetic Engineering and Biotechnology, Havana, Cuba*
Data accessibility	*In this article and in supplementary file*

## 2 Value of the data

• This data could be of interest to laboratories studying the interactions of DENV with human plasma proteins.
• Data presented can be compared with list of proteins identified by different methodologies as putative DENV binding proteins.
• Data of proteins present in the different chromatography fractions could be useful for laboratories working on the fractionation of human plasma using different chromatographic approaches.

## 3 Data

This dataset describes conditions of chromatographic fractionation of human plasma resulting in a protein sample able to modulate DENV infection in mammalian cells, provides the identity of proteins contained in the different chromatography fractions and presents data on the infection of Huh 7.5 cells with DENV2 in presence of abovementioned human plasma sample.

## 4 Experimental design, materials and methods

### 4.1 Viruses and cell lines

DENV2 (strain SB8553, isolated in Malaysia in 2002) and Huh 7.5 cells were kindly donated by Dr. Lisset Hermida (CIGB, Cuba) and Dr. Félix Rey (Pasteur Institute, France), respectively. DENV2 was propagated in Vero cells (Vero, NIBSC code 011038), determining virus titers by plaque assays in the same cell line [1]. Cells were grown in DMEM supplemented with penicillin (100 units/mL), streptomycin (100 µg/mL) and 5% and 10% (v/v) heat-inactivated fetal bovine serum (FBS) for Vero and Huh 7.5 cells, respectively.

The mouse brain-derived virus preparation was obtained by homogenization of suckling mouse brains infected with DENV2 (New Guinea strain) using RPMI-1640 medium. Purified flavivirus-reactive mAb 4G2 was provided by the Unit for Production of Monoclonal Antibodies (CIGB, Cuba).

## 4.2 Fractionation of human plasma samples

**Fig. 1:**
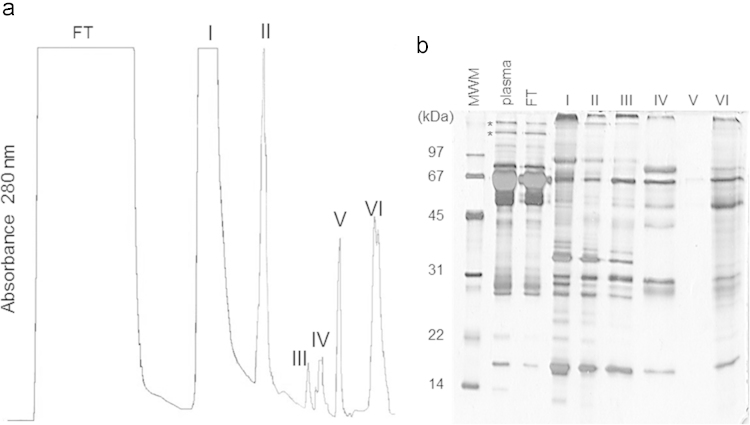
Fractionation of human plasma in DE-52 AXC. (a) Chromatographic profile. FT, flow through. Bound proteins were eluted from the column with (I) 0.3 M NaCl, (II) 0.6 M NaCl, (III) 1 M NaCl, (IV) 100 mM sodium acetate pH 3.0, (V) water and (VI) NaOH. (b) SDS-PAGE analysis of the chromatography fractions. MWM, molecular weight markers. The lane identification tags match those of the chromatogram.

Frozen human plasma samples from healthy volunteers were obtained from expired stocks of local blood banks, pooled and fractionated by anion exchange chromatography (AXC). Briefly, 125 mL of pooled human plasma were dialyzed against equilibration buffer (50 mM Hepes pH 6, 60 mM NaCl, 1 mM EDTA) and then centrifuged at 10,000 g for 30 min at 4 °C, diluted 3-fold in equilibration buffer and subjected to tandem filtration through Whatman 1 qualitative filter paper (Whatman, UK) and a 0.45 µm membrane. The resulting sample was loaded at a flow rate of 10 cm/h onto an XK-26 chromatography column (GE Healthcare, USA) packed with 10 mL of DE-52 gel, which was next washed with equilibration buffer until absorbance at 280 nm returned to baseline (bound protein represented, in average, 1.2% of the initial protein contents of the sample and no more than 60% of the total protein binding capacity estimated for DE-52 at pH 6.0). Then, the column was washed sequentially with preparations of equilibration buffer containing increasing concentrations of NaCl (0.3 M, 0.6 M and 1 M) at 30 cm/h, and the protein species still bound to the column afterwards were eluted by the successive application of 0.02 M sodium acetate pH 3.0, water and 0.01 M NaOH. Chromatography fractions were used either separately or pooled in a single sample (ELU_AXC_) [2] (Fig. 1). Upon collection, a protease inhibitor cocktail consisting of 1 μg/mL leupeptin, 1 μg/mL pepstatin A, 1 μg/mL soybean trypsin inhibitor and 1 mM PMSF was added to the eluted fractions, which were subsequently dialyzed against 10 mM Hepes pH 7.0, 0.15 M NaCl, 1 mM CaCl_2_ and stored at −20 °C until used.

## 4.3 Virus-binding activity of plasma fractions

**Fig. 2:**
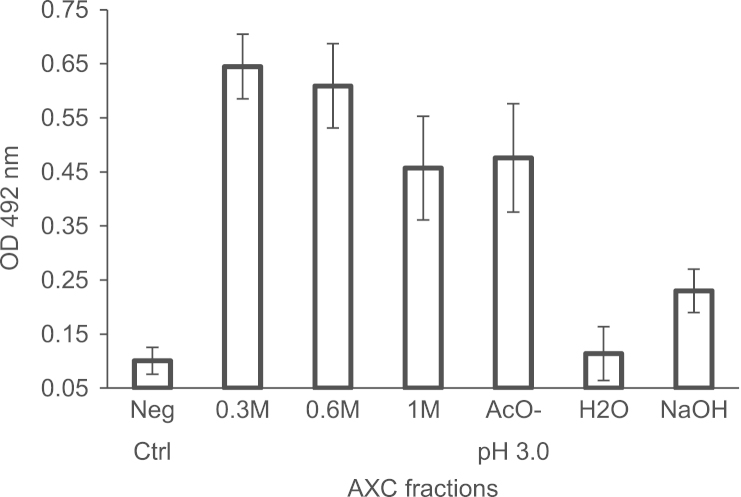
Results of the ELISA evaluating the binding of AXC fractions to DENV2. Data represent mean with range from two independent experiments, each performed in triplicate.

Microtiter plates were coated with 1 µg protein content of the corresponding AXC chromatography fraction diluted in 50 µL of 0.05 M sodium carbonate/bicarbonate buffer, pH 9.6. After coating, the plates were washed with 0.05% Tween 20 in 10 mM Hepes pH 7.0, 0.12 M NaCl, 1 mM CaCl_2_ (HBCa-T) and remaining non-specific binding sites were blocked with 1% Bovine Serum Albumin in HBCa-T for 1 h at 37 °C. Specified dilutions of mouse-derived DENV2 were added to the plates and the binding reaction was allowed to proceed for 2 h at 37 °C. Bound virus was detected by incubation with biotinylated Mab 4G2 at 5 µg/mL (1 h at 37 °C) followed by washing with HBCa-T and incubation with a streptavidin–peroxidase conjugate (1 h at 37 °C). After washing, the plates were developed by adding 0.001% H_2_O_2_, 1 mg/mL o-phenylenediamine in 0.01 mol/L citric acid-phosphate buffer pH 5.0 for 20 min at room temperature, stopping the reactions by adding 20 µL of 2.5 M H_2_SO_4_, and reading absorbance at 492 nm in a microplate reader (Fig. 2).

## 4.4 Protein composition of AXC eluates

The pH of the AXC eluates was first adjusted to 8.0 by adding 0.4 M Tris–HCl pH 8.0, 3 M urea (*i.e.* 0.3 M NaCl, 0.6 M NaCl, 1 M NaCl, AcO^−^, H_2_O and NaOH). Disulfide bonds were reduced and the resulting free thiol groups were alkylated by adding dithiothreitol to a final concentration of 10 mM and incubating samples at 37 °C for 2 h, and then adding acrylamide to a concentration of 25 mM and incubating the samples for 1 h at 25 °C. The reduced/alkylated samples were diluted with water to 0.1 M Tris/HCl pH 8.0, 0.75 M urea and sequencing grade trypsin (Promega, USA) was added to an enzyme:substrate ratio of 1:50, incubating the resulting mixture for 16 h at 37 °C. Before analysis by LC-MS/MS, all peptide mixtures were desalted in Sep-Pak tC18 cartridges (Waters, USA), eluted in 60% acetonitrile, 0.1% formic acid, concentrated by evaporation under vacuum and dissolved in 0.1% formic acid.

Peptide digest mixtures were analyzed by liquid chromatography coupled to MS experiments (LC-MS/MS), using an Ultimate 3000 RSLC nano system (Thermo Fisher Scientific, USA) connected on-line to a linear trap quadrupole (LTQ)-Orbitrap Velos mass spectrometer (Thermo Fisher Scientific). One microliter of each sample was injected into an Acclaim PepMap trap column (2 cm×75 μm i.d., C18, 3 μm, 100 Å) (Dionex, USA) at 5 µL/min, using an aqueous solution containing 0.05% (v/v) trifluoroacetic acid and 1% acetonitrile. After three minutes, the trap column was connected to an Acclaim PepMap RSLC 15 cm×75 μm i.d., C18, 2 μm, 100 Å analytical column (Dionex, USA). The peptides were eluted using a linear 2–35% gradient (solvent A: 0.1% (v/v) formic acid in HPLC-grade water; solvent B: 0.1% (v/v) formic acid in HPLC-grade acetonitrile) at 300 nL/min over 66 min, followed by a washing step of 7 min at 90% solvent B. MS spectra were acquired in the orbitrap analyzer over a mass range of 300–2000 Th. Peptide fragmentation was performed with the collision induced dissociation method at a normalized collision energy of 35%. MS/MS spectra were acquired in the linear trap.

Protein identification based on MS/MS spectra was performed by searching the Swissprot database with the Mascot ver. 2.3 engine [3]. Searching limiters included propionamide cysteine as a fixed modification as well as methionine oxidation and deamidation of glutamine and asparagine as variable modifications, a precursor ion *m*/*z* tolerance of 10 ppm and trypsin as proteolytic enzyme, with up to one missed cleavage. Only doubly- and triply-charged precursor ions were considered, using an error of 0.2 Da for matching daughter ions in the MS/MS spectra. The false discovery rate (FDR) was set to 1% for peptide and protein identifications. In the case of proteins identified from a single peptide, the ESI-MS/MS spectra were manually inspected and the identification considered reliable only if four or more consecutive C-terminal y_n_′′ fragment ions were assigned to intense signals and complementary b_n_ ions were detected.

Data of protein identification is presented in Supplementary file S1.

## 4.5 DENV2 infection in presence of ELU_AXC_

**Fig. 3:**
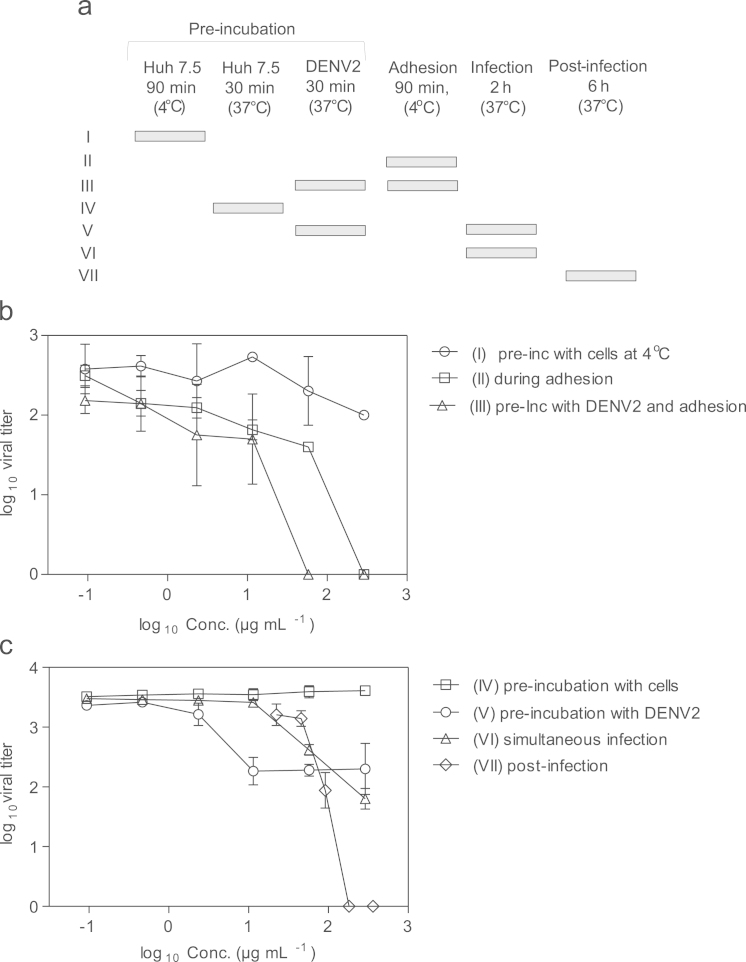
Results of DENV2 infection in presence of ELU_AXC_ preparation. (a) Schematic representation of time-of-addition experiments. Horizontal bars represent the presence of ELU_AXC_ preparation. In separated graphs are presented the results of experiments where ELU_AXC_ preparation was incubated with cells at 4 °C (b) or 37 °C (c). The roman numerals in the legends are matched to those in (a). The amount of DENV2 in 24 h p.i supernatants was determined by a plaque formation assay in Vero cells. Results represent the average of two independent experiments performed in duplicate. The error bars represent standard deviations.

Ninety-six well plates (Costar, 3597) were seeded with 3×10^4^ cells/well of the Huh 7.5 line and incubated 18–24 h at 37 °C, 5% CO_2_ until 90% confluence. The DENV2 inoculum was added at a multiplicity of infection (m.o.i) of 0.01 and infection was allowed to proceed for 2 h. Non-internalized virions were inactivated by a short treatment with Gly pH 3.0, the infection was allowed to proceed for 24 h, and viral yield was evaluated in cell supernatants by plaque formation assays in Vero cells (Fig. 3). For the assays involving a pre-incubation step at 4 °C, the monolayers were pre-chilled for 5 min on ice, the indicated dilutions of plasma sample alone or mixed with DENV2 in DMEM w/o FBS were added, and the plates were further incubated for 90 min at 4 °C. Afterwards, the monolayers were washed twice with DMEM 2% FBS (pre-chilled to 4 °C). The plates incubated with virus:plasma sample mixtures were then incubated for 24 h at 37 °C.

Post-infection activity was evaluated by infecting the cells as described above and then, after washing twice with DMEM w/o FBS, adding dilutions of the plasma samples in DMEM w/o FBS and incubating the plates for 6 h at 37 °C, 5% CO_2_. Next, the monolayers were washed again and DMEM 2% FBS was added. The supernatants were collected 24 h post infection for virus titration in a plaque assay using Vero cells.

## 4.6 Toxicity assay

**Fig. 4:**
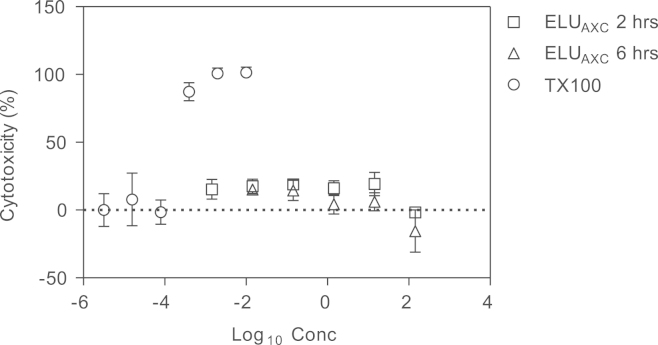
Results of acute toxicity testing. Concentration is expressed in % for TX100 concentration and µg/mL for ELU_AXC_. Data correspond to mean with range from two independent experiments, each performed in triplicate.

Vero cells were seeded in 96-well plates and treated with indicated dilutions of ELU_AXC_ in DMEM, 2% FBS either for 2 h or 6 h at 37 °C, 5% CO_2_. At the end of the incubation, cell supernatants were removed and 50 μL of 2 mg/mL 3-(4,5-dimethylthiazol-2-yl)-2,5-diphenyltetrazolium bromide (MTT) (Invitrogen, EE.UU) in PBS was added. Next, cells were incubated for 4 h at 37 °C, 5% CO_2_ before solubilization of formazan crystals in 100 µL of Dimethylsulfoxide. Absorbance was measured at 540 nm. For calculation, cell death induced by 0.1% Triton X-100 was defined as 100% (Fig. 4).

## Footnotes

Appendix A Supplementary data associated with this article can be found in the online version at 10.1016/j.dib.2015.12.016.
